# Healthcare workers safety: a cohort study using healthcare utilisation databases on vaccination and vaccine timeliness impact against SARS-CoV-2 infection

**DOI:** 10.1038/s41598-024-84100-0

**Published:** 2025-01-02

**Authors:** Edlira Skrami, Andrea Faragalli, Marica Iommi, Marco Morbidoni, Cristina Mancini, Antonella Guidi, Annalisa Cardone, Marco Pompili, Pietro Serafini, Remo Appignanesi, Luigi Ferrante, Flavia Carle

**Affiliations:** 1 Center of Epidemiology, Biostatistics and Medical Information Technology, Department of Biomedical Sciences and Public Health, Università Politecnica delle Marche, Ancona, 60126 Italy; 2 Environment, Health and Epidemiology Department, Hygiene and Public Health Service, Local Health Authority Ancona, Ancona, Italy; 3 Regional Health Agency of Marche, Ancona, Italy; 4 Control Management Department, Local Health Authority Ancona, Ancona, Italy; 5 Risk Management and Clinical Governance Unit, Local Health Authority Ascoli Piceno, Ascoli Piceno, Italy

**Keywords:** COVID-19, SARS-CoV-2, Vaccine impact, Vaccine timeliness, Healthcare workers, Healthcare Utilisation databases, Diseases, Health care, Medical research, Risk factors

## Abstract

Healthcare Workers (HCWs) are at ongoing risk of SARS-CoV-2 infection, potentially contributing to its transmission. This study assessed full vaccination and vaccination timeliness impact on SARS-CoV-2 infections among HCWs in Italy’s Marche Region, using Healthcare Utilization Databases. We evaluated vaccination coverage and its associated factors. The cohort comprised 21,118 HCWs aged 18–70 from the region’s five Local Health Authorities (LHA), enrolled between February 2020 - May 2021. Factors associated with full vaccination were assessed using multiple logistic regression. The impact of vaccination status, time to vaccination, occupational role, age, gender, and health status on infection risk was analysed with a multiple Cox regression model, adjusting for vaccination coverage velocity, swabbing probability, and monthly intensive care unit admissions rate in each LHA. Of the cohort, 81.2% were fully vaccinated. Factors associated with full vaccination included age, role, LHA, prior infection, and health status. Vaccination reduced infection risk by 77% (95% CI: 70–82). Infection risk was higher among healthcare assistants, nurses/physiotherapists/technicians compared to physicians, among male HCWs, and it decreased as vaccination timeliness increased. Vaccination timeliness is crucial for reducing SARS-CoV-2 infection risk among HCWs, regardless of their characteristics. This underscores the importance of efficiently organizing vaccination administration across different territories and for all HCW categories.

**Supplementary Information:**

The online version contains supplementary material available at 10.1038/s41598-024-84100-0.

## Introduction

Vaccines against the Severe Acute Respiratory Syndrome Corona Virus 2 (SARS-CoV-2) have been the worldwide strategy to contain its transmission. Evidence on the short-term effectiveness of these vaccines against SARS-CoV-2 infection is available from both controlled^1–4^ and real-world studies^5–12^. In Italy, the vaccination campaign started on December 27, 2020, at the end of the second wave of the SARS-CoV-2 pandemic. By that time, Italy had recorded 2,047,696 cases and 71,925 deaths^13^. The first half of 2021 was marked by the onset of the third wave, which peaked in March 2021 with 26,824 new daily cases and decreased to the lowest value at the end of June with 289 new daily cases^13^.

The initial phase of the vaccination campaign involved individuals at high risk of exposure to SARS-CoV-2 infection, such as healthcare workers (HCWs)^7^, on a voluntary basis initially, becoming obligatory by April 2021 ^14^. Vaccination followed the approved schedules indicated by the European Medicines Agency^15^ and the Italian agency (Agenzia Italiana del Farmaco, AIFA). During the first six months of the campaign, the more transmissible Alpha variant (B.1.1.7) was the predominant circulating strain in Italy^16^. In this period, reverse transcription-polymerase chain reaction (RT-PCR) and rapid antigen tests for SARS-CoV-2 were available, but a positive rapid test had to be confirmed by an RT-PCR test for COVID-19 diagnosis. Regular screening for all HCWs was expected; however, testing policies could have varied and been implemented differently throughout the territory.

The implementation of a large-scale vaccination program involves complex planning, and its effectiveness depends on the ability to quickly roll out vaccination centres, ensure vaccine availability, maintain the security of the vaccine supply, and manage logistics^17^. Possible delays in starting and completing vaccination impact coverage and infection risk, as suggested by theoretical models^18^. The extent to which vaccination timeliness impacts infection risk remains unexplored. Thus, it is crucial to understand the role of vaccination timing and to identify predictors of post-vaccine SARS-CoV-2 infection, particularly among healthcare workers who are continuously exposed to infection and play a potential significant role in its transmission, in order to plan effective priority interventions.

Furthermore, although high levels of vaccine uptake among HCWs were reached at the beginning of the vaccination campaign in various settings and countries^7,19,20^, characterizing vaccination coverage according to sociodemographic factors, health status and territorial administrative organization is needed to inform further vaccination strategies.

In this study, we aimed at evaluating the impact of full vaccination and vaccination timeliness on SARS-CoV-2 infections during the first five months of vaccine campaign among healthcare workers, using secondary sources of health data from a Region of central Italy. Specifically, we analysed vaccine effectiveness and the effect of vaccine administration timing on infection rates, taking into account demographic, health-related, and occupational factors, as well as the pandemic context. We also assessed vaccination coverage and examined demographic, health-related, and occupational factors associated with vaccination uptake among healthcare workers.

## Results

Overall, 21,118 HCWs met the inclusion criteria and were included in the study cohort. They had a median age of 49 years (1st ; 3rd quartiles: 40; 57 years), most participants were females (14340 [67.9%]), had good health conditions (14505 [69%]), and were nurses, physiotherapists, or technicians (11122 [52.7%]).

### Vaccination coverage

By May 31st, 2021, 17,155 HCWs (81.2% [95% CI: 80.7; 81.8]) were fully vaccinated. One dose of vaccine was given to 1366 participants (6.5% [95% CI: 6.1; 6.8]), of whom 776 (56.8% [95% CI: 54.1; 59.4]) did not have enough follow-up time to receive the second dose according to the vaccine type; 2596 HWs (12.3% [95% CI: 11.9; 12.3]) were unvaccinated.

Table 1 shows the distribution of the cohort characteristics according to vaccination status. Vaccinated HCWs were significantly more frequently employed in LHA2, which includes the regional highly specialized hospital treating the majority of COVID-19 patients. Those partially vaccinated with sufficient time to receive full vaccination were significantly more frequently healthcare assistants and had a higher probability of a prior SARS-CoV-2 infection compared to the other groups. Unvaccinated HCWs were significantly more likely to be under fifty years old and to work in the southern part of the Region (LHA4 and LHA5) compared to vaccinated and partially vaccinated HCWs. They also had more frequently good health conditions (MCS score equal to 0) and held administrative or other roles compared to vaccinated and partially vaccinated HCWs with sufficient time to receive full vaccination.

**Table 1:** Characteristics of the cohort members (*n* = 21118) according to the vaccination status.

Characteristics		Total		Fully vaccinated		Partially vaccinated and enough FU		Partially vaccinated and no FU^a^		Unvaccinated		*p*
		*n*	%	*n*	%	*n*	%	*n*	%	*n*	%	
Total		21,118	100	17,155	81.23	590	2.79	776	3.68	2597	12.3	
Age classes	< 50	11,233	53.19	8969	52.28	316	53.56	402	51.80	1547	59.57	< 0.001^b^
	≥ 50	9885	46.81	8187	47.72	274	46.44	374	48.20	1050	40.43	
Gender [Female]		14,340	67.90	11,653	67.93	393	66.61	525	67.65	1769	68.12	0.911^b^
MCS	0	14,505	68.69	11,691	68.15	399	67.63	562	72.42	1853	71.35	0.004^b^
	1–4	5216	24.70	4297	25.05	159	26.95	172	22.17	588	22.64	
	≥ 5	1397	6.61	1167	6.80	32	5.42	42	5.41	156	6.01	
Occupational role	Physician	5480	25.95	4514	26.31	151	25.59	140	18.04	675	25.99	< 0.001^b^
	Nursing/Physiotherapist/Technician	11,122	52.67	9162	53.41	310	52.54	428	55.16	1222	47.05	
	Healthcare assistant	1648	7.80	1289	7.51	70	11.87	65	8.38	224	8.63	
	Administrative	1789	8.47	1366	7.96	41	6.95	85	10.95	297	11.44	
	Other	1079	5.11	824	4.81	18	3.05	58	7.47	179	6.89	
Local health authority workplace (LHA)	LHA1	4404	20.85	3377	19.69	159	26.95	241	31.06	627	24.14	< 0.001^b^
	LHA2^c^	8134	38.52	6834	39.84	199	33.73	275	35.44	826	31.81	
	LHA3	3695	17.50	3092	18.02	115	19.49	123	15.85	365	14.05	
	LHA4	1840	8.71	1443	8.41	42	7.12	55	7.09	300	11.55	
	LHA5	2782	13.17	2231	13.00	68	11.52	70	9.02	413	15.91	
	NA	263	1.25	178	1.04	7	1.19	12	1.54	66	2.54	
Prior SARS-CoV-2 infection, cumulative probability (95% CI)^d^		0.08	0.07; 0.08	0.06	0.05; 0.06	0.46	0.41; 050	0.25	0.21; 0.28	0.11	0.10; 0.12	< 0.001^e^
Vaccine type	Pfizer: BNT162b2	17,786	84.22	16,888	98.44	578	97.97	320	41.24	-		< 0.001^f^
	Moderna: mRNA-1273	378	1.79	235	1.37	12	2.03	131	16.88	-		
	AstraZeneca: ChAdOx1	351	1.66	26	0.15	-		325	41.88	-		
	Janssen: Ad26.COV2.S	6	0.03	6	0.04	-		-		-		

CI: Confidence Interval; FU: follow up; NA: not available; MCS: Multisource Comorbidity Score; ^a^ participants without enough follow-up (FU) time to observe the second dose; ^b^ Chi-square test; ^c^ LHA2 includes the regional high specialized hospital treating the majority of Covid-19 patients; ^d^ Kaplan-Meier method; ^e^ Log Rank test; ^f^ Fisher exact test.

Most of the vaccinated and partially vaccinated HCWs (96%) received the Pfizer vaccine. Among partially vaccinated HCWs who did not have enough time to receive full vaccination, the AstraZeneca and Moderna vaccines were administered significantly more frequently than in the other groups.

### Factors associated with vaccination

Table 2 shows the results of the logistic regression analysis. Factors significantly associated with vaccination were age, professional role, LHA of the workplace, prior SARS-CoV-2 infection, and health conditions. Specifically, the probability of being fully vaccinated was 23% higher among HCWs aged 50 years or older compared to those younger than 50. It decreased by 22%, 41%, and 33% for healthcare assistants, administrative staff, and other staff, respectively, compared to physicians. The probability was 48% higher for those working in the LHA2 and LHA3 and 15% higher for those working in the LHA5 compared to LHA1. A prior SARS-CoV-2 infection reduced the probability of vaccination by 74% compared to HCWs not previously infected. In contrast, the probability of being fully vaccinated increased by 11% for HCWs with MCS scores between 1 and 4 and by 18% for HCWs with MCS scores of 5 or higher compared to those with MCS scores of zero. Gender was not associated with the probability of being fully vaccinated.

**Table 2:** Factors associated with vaccination coverage (fully vaccinated vs. unvaccinated and partially vaccinated). Results of the multiple logistic regression analysis.

Variables	OR	95% CI	*p*
Age classes (ref. < 50 years): ≥ 50 years	1.23	1.14; 1.32	< 0.001
Gender (ref. Female): Male	0.99	0.91; 1.07	0.740
LHA (ref. 1)			
2	1.48	1.35; 1.63	< 0.001
3	1.48	1.32; 1.66	< 0.001
4	1.04	0.91; 1.19	0.552
5	1.15	1.02; 1.30	0.002
Role (ref. Physician)			
Nurse/Physiotherapist/Technician	0.98	0.89; 1.07	0.987
Healthcare assistant	0.78	0.68; 0.91	0.002
Administrative staff	0.59	0.51; 0.67	< 0.001
Other staff	0.66	0.56; 0.78	< 0.001
Prior SARS-CoV-2 infection (ref. No): Yes	0.26	0.23; 0.29	< 0.001
MCS (ref. 0)			
1–4	1.11	1.02; 1.21	0.016
≥ 5	1.18	1.01; 1.38	0.038

CI: Confidence Interval; LHA: Local Health Authority; MCS: Multisource Comorbidity Score; OR: Odds-Ratio; Likelihood Ratio-test: p-value < 0.001; Hosmer–Lemeshow test: p-value = 0.06.

### Impact of vaccination on the risk of infection

During the follow-up period (median of 155 days), among HCWs who were swabbed at least once (*n* = 7309), we observed 103 SARS-CoV-2 infections in the vaccinated group (*n* = 6099) and 624 in the unvaccinated group (*n* = 1210). One death occurred between December 27, 2020, and May 31, 2021, in the unvaccinated group. Table 3 shows the results of the Cox regression analysis. Vaccination significantly reduced the risk of infection by 77% (95% CI: 70%; 82%) compared to unvaccinated individuals (Schoenfeld residual test: *p* = 0.911). Although the evaluation was conducted on a subset of the total sample, the post-analysis power of the impact of vaccination on the risk of infection is 1, according to the formula by Wang, Zhang and Lu (2014)^21^ using the parameters estimated by the Cox model.

**Table 3:** Factors associated with the SARS-CoV-2 infection. Results of the multiple Cox regression analysis.

Variables	HR	LL	UL	*p*
Vaccinated (ref. no): Yes	0.23	0.18	0.30	< 0.001
Role (ref. Physician)				
Healthcare assistant	1.66	1.22	2.27	0.001
Nursing/Physiotherapist/Technician	1.37	1.09	1.72	0.006
Age (ref. < 50 years): ≥ 50 years	1.15	0.96	1.38	0.130
Gender (ref. Female): Male	1.45	1.20	1.75	< 0.001
MCS (ref. 0)				
1–4	1.03	0.84	1.27	0.744
≥ 5	0.94	0.64	1.38	0.754
Daily probability of being swabbed	0.99	0.92	1.06	0.744
Rate of monthly hospital admissions in ICU	1.20	0.99	1.45	0.061
Vaccination coverage velocity^a^	1.16	1.07	1.26	< 0.001

ICU: intensive care unit; HR: Hazard Ratio; LL: Lower Limit; MCS: Multisource Comorbidity Score; UL: Upper Limit; ^a^Vaccination coverage velocity: days required for 65% of each Healthcare Workers category to complete the vaccination cycle in each Local Health Authority (rank transformed); Wald test = 188, df = 14, *p* < 0.001; VIF (Variance Inflation Factor) < 3.

The risk of infection was significantly higher for HCWs in healthcare assistant roles and for nurses/physiotherapists and technicians compared to those in physician roles, with increased risks of 66% (95% CI: 22%; 127%) and 37% (95% CI: 9%; 72%), respectively. Additionally, males had a 45% (95% CI: 20%; 75%) higher risk of infection compared to females. Health condition status was not associated with the risk of infection. Among the factors used to characterize the LHAs, vaccination coverage velocity was the only one significantly associated with infection risk (HR: 1.2, 95% CI: 1.1; 1.3).

**Fig. 1:**
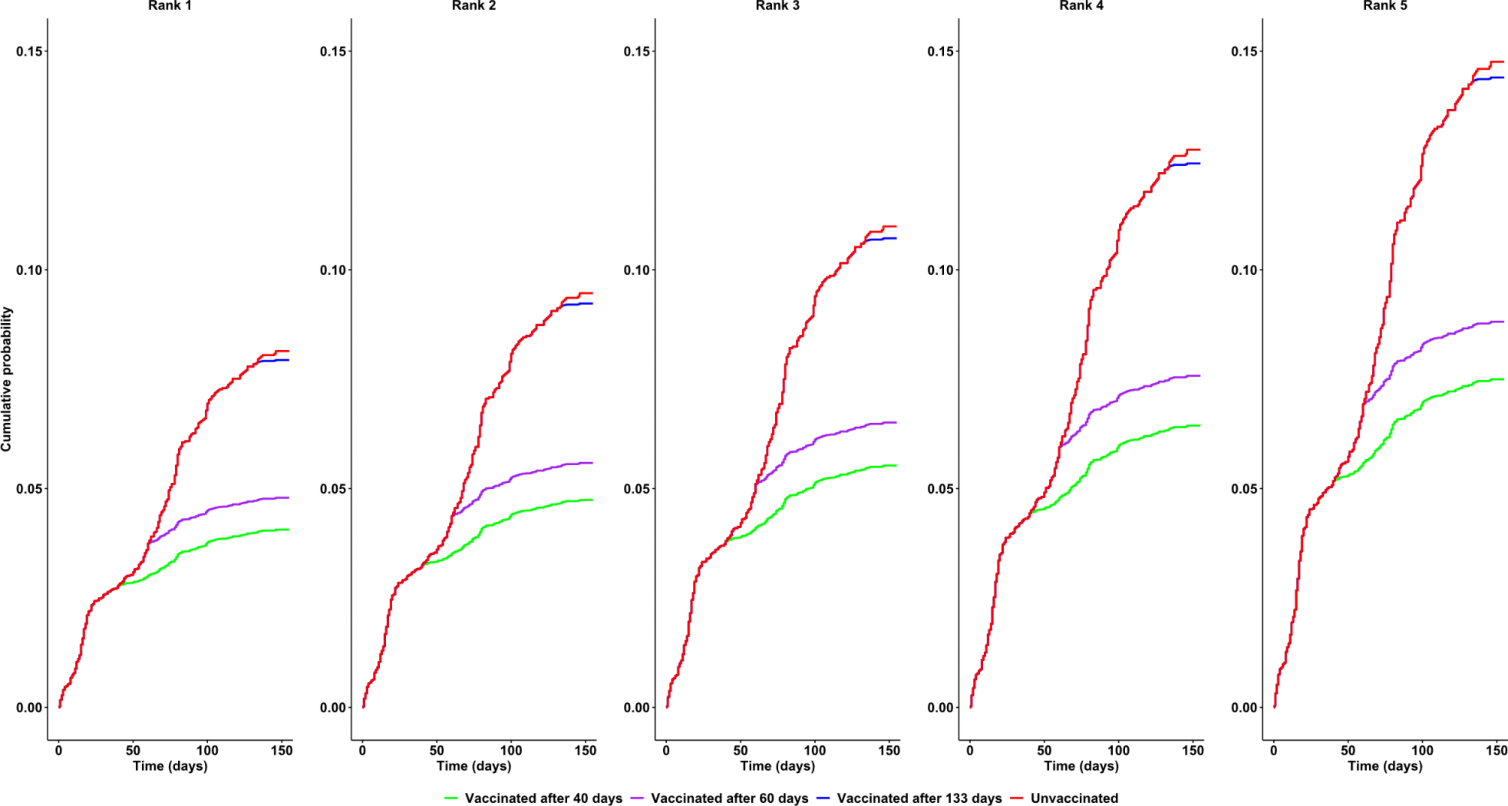
Cumulative probability of infection according to vaccination speed in male physician. Rank 1 corresponds to the highest vaccination coverage velocity (days required for 65% of each Healthcare Workers category to complete the vaccination cycle, 39 days), Rank 2 (41 days), Rank 3 (54 days), Rank 4 (67 days), while Rank 5 corresponds to the lowest vaccination coverage velocity among Local Health Authorities (69 days).

Figure 1 shows the cumulative probability of infection estimated from the Cox regression model for male physicians, stratified by the rank of LHAs’ vaccination coverage velocity. As expected, the vaccination timeliness reduces the probability of infection. This probability increased as LHAs’ vaccination coverage velocity decreased, moving from those with higher to those with lower vaccination coverage velocity (ranked from 1 to 5, respectively). The same pattern of risk reduction was observed across all occupational roles and genders (Supplementary Table S1). Infection probability also increased with the time elapsed since the start of the vaccination campaign. For example, for a female healthcare assistant in a geographical area with a vaccination coverage velocity rank of three, the infection probability increased from 6.5% (95% CI: 5.4; 7.5) to 7.6% (95% CI %: 6.5; 8.8) to 12.5% (95% CI: 11.1; 13.9) depending on whether her vaccination occurred at 40, 60, or 133 days after the start of the vaccination campaign, respectively. If she was not vaccinated, the infection probability was estimated to be 12.8% (95% CI: 9.8; 15.8).

## Discussion

This large cohort study of healthcare workers in a region of central Italy contributes to enhance the knowledge about Healthcare Workers safety by providing a precise and real-world quantification of how vaccination timeliness impacts the risk of SARS-CoV-2 infection, a topic that had not been previously explored. In addition, the study’s use of multiple secondary data sources, covering a well-defined and unselected population, allowed for a comprehensive examination of various determinants of vaccination coverage and infection risk, by tracing the health history of HCWs, pandemic dynamics, and local strategies for coping with the emergency. The study demonstrates the benefit of rapid full vaccination, regardless of vaccine type, in reducing the risk of SARS-CoV-2 infection by 77% during the first five months of the vaccination campaign, starting 7 days after vaccination. We found that this reduction in infection risk was associated with vaccination timeliness, while accounting for other relevant covariates such as occupational position, demographic characteristics, health conditions, and territorial characteristics related to the pandemic. Our results highlight the importance of efficiently organizing and administering vaccination homogeneously across both geographical areas and health worker categories.

### Vaccination coverage and associated factors

Five months after the vaccine campaign began, over 81% of healthcare workers had completed their vaccination. Similar levels of vaccination coverage among HCWs during the early months of the vaccine campaign were reported in other Italian studies^19,20^ and could be considered high, given that vaccination only became mandatory for HCWs in Italy in April 2021 ^14^. Indeed, we observed a vaccination coverage of 74.4% just before this mandatory period (March 31, 2021, data not shown). Disparities in vaccination coverage across the Region likely reflect organisational differences in the vaccine distribution plan, as further shown when evaluating the risk of infection. While vaccination started almost simultaneously in all LHAs, the pace of progress differed. Despite prioritizing HCWs for vaccination, our results indicated that vaccination coverage was characterized by high variability, particularly across age groups and occupational roles, consistent with findings from other studies^20,22–24^. Analysing the determinants of vaccine uptake is crucial for refining vaccination strategies, as continuous booster doses are needed to protect against new variants of concern and because vaccine immunity wanes over time^22,25–27^. Additionally, our study corroborates evidence from Bedston et al.^22^ in a national Welsh cohort, showing that health profile vulnerability was associated with a higher probability of being vaccinated. The lower probability of being vaccinated among infected HCWs aligns with Italian guidelines^28^.

### Impact of vaccination on SARS-CoV-2 infection

The study showed a clear impact of vaccines on the infection risk in healthcare workers. Although we considered all available vaccines during the study period, over 98% of the vaccinated HCWs in the cohort received the Pfizer vaccine. The results pertain to all regional healthcare settings during a period with a weekly incidence of SARS-CoV-2 infection ranging between 41.9 and 319 per 100,000 inhabitants^13^. The reduction in the infection risk found in our study during a period characterized by the dominance of the Alpha variant is consistent with a previous Italian cohort study^19^ and also a meta-analysis on vaccine effectiveness based on 290 studies from different countries^29^. However, as discussed by Hall et al.^24^ and Pople et al.^24,30^, comparing these results with those of other studies remains challenging due to non-overlapping evaluation periods and differences in study population and design, screening strategies, definitions of exposure and outcome, and the analytical methods used.

To the best of our knowledge, only Pople et al.^30^, in a multicentre cohort study of HCWs in England, estimated the effect of vaccine timing on SARS-CoV-2 risk. Using a unique methodology, our study brings additional real-world evidence to the literature demonstrating the role of vaccine timeliness in reducing the probability of infections. Efforts should be made not only at the national level but also at the local level to increase health system capacity to sustain rapid immunisation coverage in response to the evolution of the pandemic.

Consistent with previous studies^22,24,30,31^, occupation was a risk factor for SARS-CoV-2 infection. Healthcare assistants, nurses, physiotherapists, and technicians have a higher risk of infection than physicians, regardless of vaccination status and health condition. Exploring the number and type of contact between these categories and patients, as well as the use of protective equipment, could help to better understand exposure to COVID-19 and to optimise strategies to reduce infection spread.

Male gender was also associated with a higher risk of SARS-CoV-2 infection. This finding aligns with studies performed among HCWs in Wales^22^ and in the general population, as reported by Sieurin et al.^32^ and Abate et al.^33^.

Unlike other studies, we included a factor expressing the vaccination coverage velocity in our analysis to better understand the local management of the vaccination campaign. We found that the impact of vaccination on infection risk differed between LHAs, which was related to the varying vaccination coverage velocity among them, thus reflecting disparities in organizational efforts to prevent infections. Our results underline the importance of having a healthcare organizational system prepared to deal with infectious emergencies and the necessity of evaluating the efficiency of such systems in different local contexts.

### Limitations and strengths of the study

Our study has some limitations. First, the estimation of the impact of vaccines on the risk of infection could have been influenced by nonpharmaceutical interventions, such as surgical and nonsurgical masks, gloves, goggles, face shields, gowns, and N95 masks used during the evaluation period. However, these measures were consistent throughout the region. Moreover, it is less likely that the impact of such measures provided differential protection for vaccinated versus unvaccinated HCWs. Second, the testing probability in unvaccinated HCWs was higher than in vaccinated HCWs (Supplementary Figure S1); therefore, infections were more likely to be identified among the unvaccinated HCWs, potentially biasing the impact of vaccination by overestimating the reduction in the risk among vaccinated HCWs. Third, due to the observational nature of the study, estimates may have been affected by differences between vaccinated and unvaccinated HCWs. Although the estimates were adjusted for several variables, possible residual confounding factors might have impacted the results. Lastly, the potential underestimation of total SARS-CoV-2 infections should be acknowledged, as our data relies on swabbed HCWs. Asymptomatic individuals, who are less likely to be swabbed, may have further contributed to the underreporting of SARS-CoV-2 infections.

The study has important strengths. It is a large cohort study based on the use of Healthcare Utilisation Databases with unselected regional population coverage, reflecting real-world healthcare practices and increasing the external validity of the findings to systems with similar healthcare and population structures. It extends the evidence on COVID-19 vaccination by evaluating the impact of vaccine timeliness on infection risk. Moreover, the study included previously infected HCWs, avoiding the underestimation of this category’s proportion.

### Conclusions

Vaccination timeliness is an indispensable strategy to reduce the impact of SARS-CoV-2 infection among healthcare workers, and it should be combined with achieving high vaccination coverage. National and regional healthcare systems should prepare appropriate and efficient epidemic/pandemic plans, including organizational guidelines to ensure consistent vaccination timeliness across all territories. The higher risk of SARS-CoV-2 infection among healthcare assistants, nurses, physiotherapists, and technicians compared to physicians suggests the need for further studies on risk determinants to optimise booster vaccination programs for HCWs. Additional observational studies on SARS-CoV-2 vaccine effectiveness based on Healthcare Utilisation Databases will be able to confirm and extend the results of this study, providing important evidence to support healthcare governance.

## Methods

### Study design and participants

A prospective cohort study was performed among HWs of the Marche Region, in central Italy, which has a population of 1,498,236 residents. Data on cohort members were collected retrospectively and referred to the period between January 1st, 2018 (to assess health status prior to cohort entry), and May 31st, 2021 (the most recent data available at the time of the study). Eligibility criteria for the study cohort included: being a HCW from one of the five Local Health Authorities (LHA) of the Region (Supplementary Figure S2), being aged between 18 and 70 years at the time of cohort entry, being a beneficiary of the Italian National Health Service (NHS) and a resident of the Marche Region on or before January 1st, 2018.

Cohort entry was defined as February 25, 2020 (the beginning of the pandemic, corresponding to the date of the 1st COVID-19 case in the region) for current workers, or the date of employment by March 31st, 2021 (the last update of the Healthcare Worker database of the Regional Health Authority at the time of the study) for new hires in one of the five LHAs. The study design, cohort selection, and timelines for vaccination coverage and the impact of vaccination on SARS-CoV-2 infection are illustrated in the flow diagram in Supplementary Figure S3.

### Data sources

The study used healthcare utilization databases from the Marche Region, which contain information on NHS beneficiaries, including demographic and administrative data, discharge diagnoses from both public and accredited private hospitals, and drug prescriptions^34^. Specifically, the following regional healthcare databases were used: Beneficiaries’ database, which includes the identification code, dates of birth and death, gender, and dates of assistance start and end; Hospital Discharges database, which provides information on admission and discharge dates, primary and up to five secondary diagnoses, and up to six interventions (coded using International Classification of Diseases, 9th Revision Clinical Modification, ICD-9-CM); Drug prescriptions database, which contains drug prescriptions (coded according to the Anatomical Therapeutic Chemical, ATC classification system) reimbursed by the NHS.

In addition, the regional Swab COVID-19 surveillance database and the vaccine database were used. The swab database records the date and results of RT-PCR or rapid antigen tests, while the vaccine database includes details on the type, dose, and date of vaccine administration. The Healthcare Worker database of the Regional Health Authority, which includes work start and end dates, occupational role, and the Local Health Authority workplace was also used for this study.

In compliance with current personal data protection regulations in Italy, these databases were linked through a deterministic procedure based on the beneficiary’s identification code. Data from all sources were extracted on June 22, 2021.

### Vaccination coverage

The vaccination campaign in the Marche Region began on December 27, 2020 and became mandatory for HCWs in Italy in April 2021 ^14^. Initially, the Pfizer-BioNTech vaccine was used (approved by the Italian Drug Agency on December 22, 2020, requiring two doses administered 21 days apart), followed by the Moderna vaccine (approved on January 7, 2021, requiring two doses administered 28 days apart), the Oxford-AstraZeneca vaccine (approved on January 30, 2021, requiring two doses administered 78 days apart) and the Janssen vaccine (approved on March 12, 2021, requiring a single dose). Four categories of vaccination status were identified: (1) fully vaccinated, individuals who received both doses of the same vaccine (Pfizer-BioNTech, Moderna or Oxford-AstraZeneca) or one dose of Janssen vaccine; (2) partially vaccinated with sufficient follow-up, individuals who received the first dose of a two-dose vaccine (Pfizer-BioNTech, Moderna, Oxford-AstraZeneca) and had enough follow-up time to receive the second dose according to the vaccine-specific schedule; (3) partially vaccinated without enough follow-up, individuals who received the first dose of a two-dose vaccine but had less follow-up time than required between doses, with the study ending before they received the second dose; and (4) unvaccinated, individuals who did not receive any vaccine doses.

Vaccination coverage was evaluated as the proportion of HCWs in each vaccination status category between December 27, 2020, and May 31, 2021, among cohort members.

### Impact of vaccination on SARS-CoV-2 infection

The impact of vaccination on SARS-CoV-2 infection was evaluated by comparing the excess risk of infection in unvaccinated HCWs to that in vaccinated HCWs. Partially vaccinated HCWs were considered unvaccinated until the date of their first dose administration. SARS-CoV-2 infection was defined as a positive RT-PCR test, regardless of symptom status, or as a hospital discharge with a COVID-19 diagnosis^35,36^ if it occurred before the test. To focus on infections related to HCW working status, we considered infections occurring 21 days after the date of employment^7^. To evaluate the vaccine impact, infections occurring 7 days after full vaccination were considered^7^; infections occurring within 7 days from the second dose were attributed to the unvaccinated status.

For this evaluation, only participants in the roles of physician, nurse/physiotherapist/technicians and healthcare assistants were included, comprising 87% of the cohort, as these categories are more exposed to COVID-19. By excluding administrative staff, who often work in different buildings, and other non-clinical staff such as electricians and plumbers, we ensure that our findings are specifically relevant to those directly involved in patient care, providing a clearer and more accurate assessment of vaccination effectiveness among high-risk groups. Moreover, to control for possible differences in the timing and modalities of SARS-CoV-2 testing strategies throughout the territory, the impact of vaccination on SARS-CoV-2 infection was evaluated by selecting only participants with at least one RT-PCR test during the evaluation period (December 27, 2020, and May 31st, 2021).

Follow-up began on December 27, 2020, or on the date of employment if later, or 90 days after the date of the RT-PCR positive test for HCWs with a previous infection^24,37,38^, considering them to be at risk of a new infection after this timeframe, in order to control for the different vaccination strategies and the natural immunity in HCWs with a prior infection. Participants were followed up until the date of SARS-CoV-2 infection, the termination date of employment, seven days after the date of being fully vaccinated for participants with a follow-up period of less than 21 days after full vaccination (considered the minimum period to appreciate vaccine impact on infection risk^7^), the date of the first dose administration for those partially vaccinated, death, or May 31st, 2021, whichever occurred first. In an attempt to avoid exposure misclassification, follow-up was censored at the date of the second dose if participants had no recorded data on the first dose or if they received the second dose with an interval time between doses different from the approved vaccine-specific schedule.

### Statistical analysis

Variables were summarized using absolute and percentage frequencies for qualitative variables, and median and interquartile range for quantitative variables. Comparisons between variable distributions at cohort entry according to the vaccination group, were performed using the chi-squared test (or Fisher’s exact test in cases of expected frequencies lower than five). Vaccination coverage was evaluated as point and 95% Confidence Interval (CI) estimates using the binomial distribution.

Factors associated with the probability of being fully vaccinated were investigated using a multiple logistic regression model. The vaccination status (fully vaccinated vs. unvaccinated and partially vaccinated) was considered the dependent variable; the independent variables included age (grouped: <50 years; ≥50 years), gender, LHA of the workplace, occupational role, previous infection occurring before the beginning of the vaccination campaign, and health condition measured by the Multisource Comorbidity Score (MCS)^39,40^ in the previous two years to cohort entry (considered in classes: good health condition for MCS score = 0; moderate health condition for MCS score between 1 and 4; poor health condition for MCS score ≥ 5). The likelihood ratio (LR) and Hosmer-Lemeshow (HL) tests were used to evaluate the model’s goodness of fit.

Kaplan-Meier curves were used to assess the cumulative probability of being swabbed during the follow-up according to the LHA of the workplace. Comparisons between unvaccinated and vaccinated curves stratified by LHA of the workplace were performed using the log-rank test.

Multiple Cox regression was performed to estimate the impact of vaccination on the risk of infection. Vaccination status was entered in the model as a time-dependent variable, with participants starting follow-up as unvaccinated and moving from unvaccinated to vaccinated status seven days after receiving the second dose; the proportional hazard assumption was tested using Schoenfeld residuals test. Vaccination status, occupational role, age, gender and MCS classes were entered into the model as independent variables.

To account for differences between LHAs in organizational aspects of vaccination, screening strategies, and pandemic spread, LHAs were characterized using the following metrics: (A) vaccination coverage velocity, defined as the number of days required for 65% of each HCW category to complete the vaccination cycle, calculated as the difference between the campaign start date and the full vaccination date, and then transformed into ranks. A higher rank indicates a slower vaccination coverage velocity, meaning more days were required for 65% of each HCW category to complete the vaccination cycle. The 65th percentile was the highest percentile of vaccination completion observed among all HCW categories and LHAs; (B) daily probability of being swabbed; (C) rate of monthly hospital admissions in the intensive care unit, evaluated for the entire LHA population.

The Cox model was adjusted for all the aforementioned LHAs characteristics considered as time-dependent covariates. Cox regression model accommodates time-varying covariates, which is essential given the evolving nature of the pandemic and vaccination rollout. Recognizing that the frequency of nasopharyngeal swabs, vaccine uptake, and the overall dynamics of SARS-CoV-2 infection vary over time, these aspects were incorporated into the Cox model to account for such variations. The model’s goodness of fit was assessed using the Wald chi-square test, and collinearity diagnostics were performed using the determinant of the covariance matrix of the Cox regression estimates.

Considering that the infection probability of an individual changes with modifications in vaccination status and that the duration spent in each status varies among individuals, we estimated the cumulative probability of infection in each LHA, characterized by the vaccination coverage velocity, using the parameters estimated by the Cox regression model described earlier. We explored several scenarios by combining different modalities of the model’s variables with varying times to vaccination completion. Specifically, results were generated for scenarios with vaccination completion times of 40 days, 60 days and 133 days after the start of the vaccination campaign. These times were categorized as short, medium, and long, respectively, with the latter representing the maximum follow-up period in the study (from the start of the vaccination campaign to 21 days before the end of the study). Statistical significance was assessed at a 5% probability level. All analyses were conducted using R statistical package 4.2.1 ^41^.

## Electronic supplementary material

Below is the link to the electronic supplementary material.

Supplementary Material 1

## Data Availability

The data that support the findings of this study are available from the Regional Health Agency of Marche (ARSMarche), but restrictions apply to the availability of these data, which were used under license for the current study, and so are not publicly available. Data are however available from the authors upon reasonable request and with permission of the Regional Health Agency of Marche (contact person: Marco Pompili - marco.pompili@regione.marche.it).
