# Supplementary material for: Healthcare workers safety: a cohort study using healthcare utilisation databases on vaccination and vaccine timeliness impact against SARS-CoV-2 infection
Source: Sci Rep. 2025 Jan 2;15:162. doi: 10.1038/s41598-024-84100-0 (PMC11695641; doi:10.1038/s41598-024-84100-0)
Supplement: Supplementary file 1 — Supplementary Material 1 [file 41598_2024_84100_MOESM1_ESM.docx]

**Supplementary Information**

**Figure S1.** Cumulative probability of being swabbed in each Local Health Authority (LHA) of workplace between Vaccinated and Unvaccinated Healthcare Workers.

**LHA1** **LHA2** **LHA3** **LHA4** **LHA5**


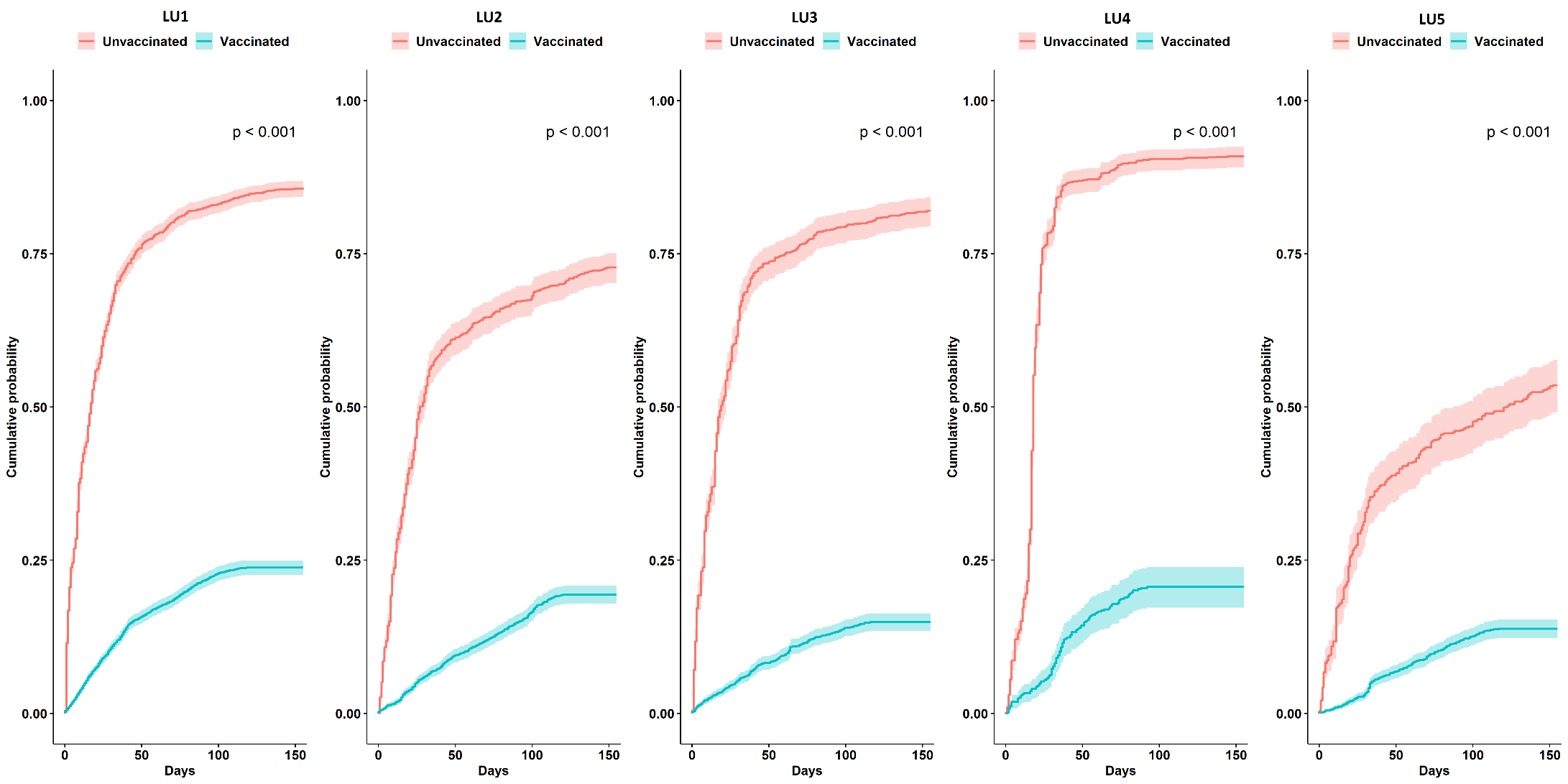


p: log-rank test.

**Figure S2.** Geographical distribution of the Local Health Authorities in Marche Region, Italy


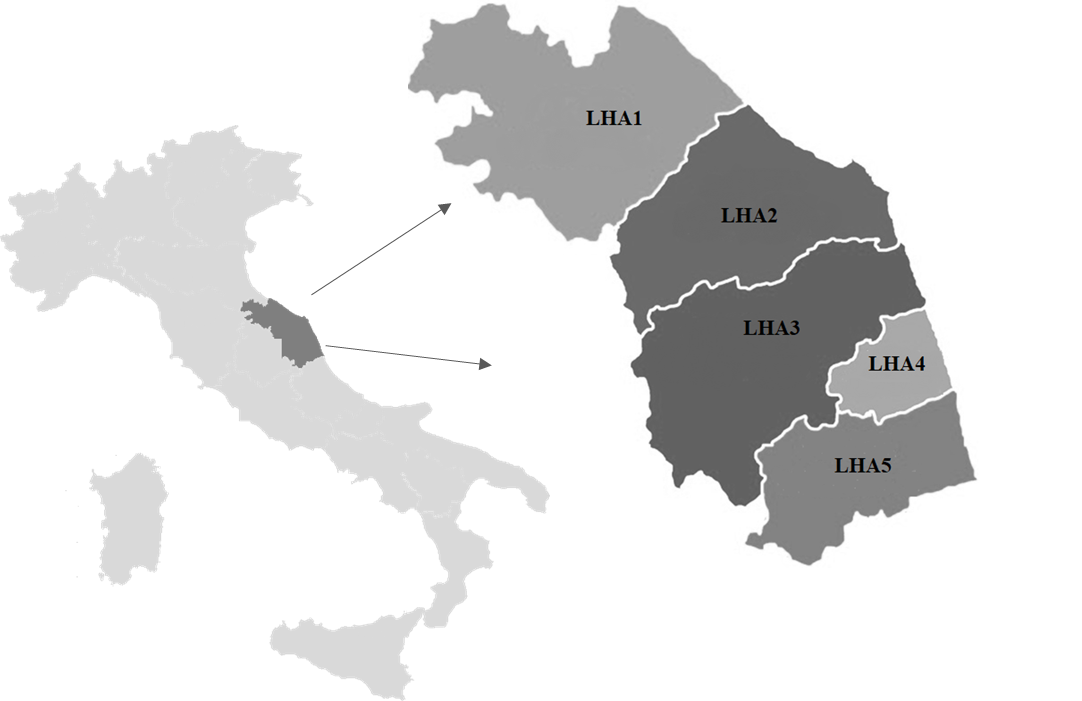


LHA: Local Health Authorities in Marche Region; the LHA2 includes the regional high specialized hospital treating the majority of Covid-19 patients.

**Source**: Modified map obtained from: <https://www.asur.marche.it/area-vasta-2?p_p_id=110_INSTANCE_jQKOAX8b10F6&p_p_lifecycle=0&p_p_state=normal&p_p_mode=view&p_p_col_id=column-2&p_p_col_pos=1&p_p_col_count=2&_110_INSTANCE_jQKOAX8b10F6_struts_action=%2Fdocument_library_display%2Fview_file_entry&_110_INSTANCE_jQKOAX8b10F6_fileEntryId=1036943>. Last access January 18, 2023.

**Figure S3.** Flow-diagram of the study design: cohort entry (February 25, 2020 to March 31, 2021) and criteria; follow-up (December 27, 2020 to May 31, 2021) period for the evaluation of the impact of vaccination on SARS-CoV-2 infection; time point for the evaluation of vaccination coverage (May 31, 2021).


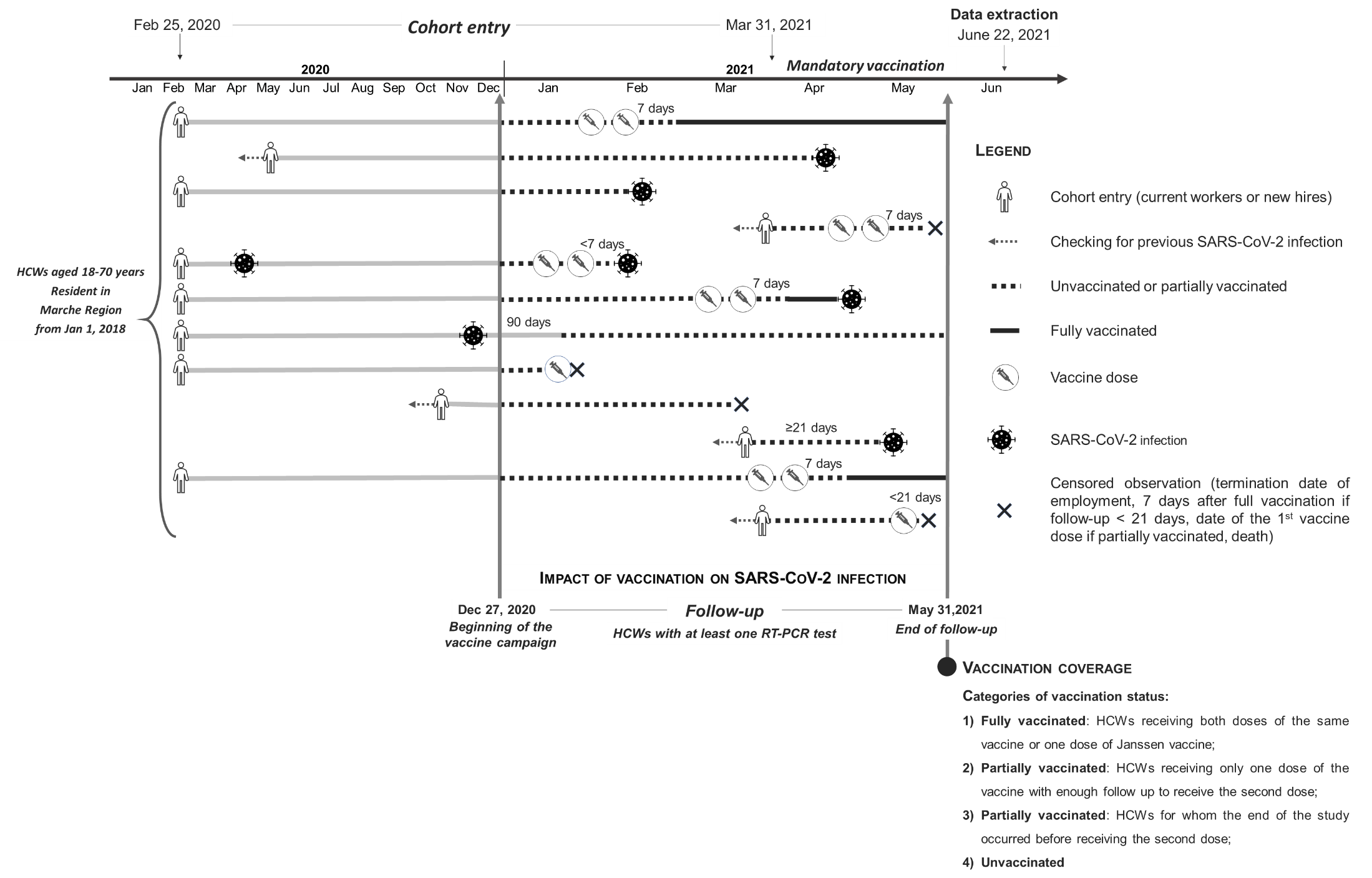


**Table S1.** Cumulative probability of infection according to vaccination coverage velocity .

|  | Vaccination coverage velocity rank | | | | | | | | | |
| --- | --- | --- | --- | --- | --- | --- | --- | --- | --- | --- |
|  | Rank 1 | | Rank 2 | | Rank 3 | | Rank 4 | | Rank 5 | |
|  | p | 95% CI | p | 95% CI | p | 95% CI | p | 95% CI | p | 95% CI |
| Physician/Male |  |  |  |  |  |  |  |  |  |  |
| Vaccinated after 40 days | 4.06 | (3.38-4.74) | 4.74 | (4.01-5.46) | 5.53 | (4.72-6.33) | 6.44 | (5.49-7.39) | 7.50 | (6.31-8.68) |
| Vaccinated after 60 days | 4.79 | (4.06-5.52) | 5.59 | (4.80-6.36) | 6.51 | (5.63-7.38) | 7.58 | (6.55-8.60) | 8.82 | (7.55-10.07) |
| Vaccinated after 133 days | 7.94 | (7.01-8.86) | 9.23 | (8.21-10.25) | 10.72 | (9.56-11.87) | 12.43 | (11.09-13.76) | 14.40 | (12.80-15.96) |
| Unvaccinated | 8.14 | (5.61-10.62) | 9.47 | (6.92-11.95) | 10.99 | (8.3-13.61) | 12.75 | (9.63-15.76) | 14.76 | (10.83-18.52) |
| Physician/Female |  |  |  |  |  |  |  |  |  |  |
| Vaccinated after 40 days | 2.82 | (2.34-3.30) | 3.29 | (2.78-3.80) | 3.84 | (3.27-4.42) | 4.49 | (3.80-5.17) | 5.23 | (4.37-6.09) |
| Vaccinated after 60 days | 3.33 | (2.81-3.84) | 3.89 | (3.33-4.44) | 4.53 | (3.91-5.16) | 5.29 | (4.54-6.03) | 6.16 | (5.24-7.08) |
| Vaccinated after 133 days | 5.54 | (4.88-6.20) | 6.46 | (5.73-7.19) | 7.52 | (6.68-8.35) | 8.75 | (7.77-9.71) | 10.16 | (8.99-11.32) |
| Unvaccinated | 5.69 | (4.00-7.35) | 6.63 | (4.94-8.28) | 7.71 | (5.92-9.47) | 8.97 | (6.86-11.03) | 10.42 | (7.70-13.07) |
| Nurse-Physiotherapist-Technician/Male | | |  |  |  |  |  |  |  |  |
| Vaccinated after 40 days | 5.54 | (4.65-6.43) | 6.46 | (5.53-7.38) | 7.52 | (6.50-8.53) | 8.75 | (7.55-9.93) | 10.16 | (8.67-11.64) |
| Vaccinated after 60 days | 6.53 | (5.57-7.47) | 7.60 | (6.59-8.60) | 8.84 | (7.73-9.94) | 10.27 | (8.97-11.56) | 11.92 | (10.31-13.49) |
| Vaccinated after 133 days | 10.75 | (9.53-11.95) | 12.47 | (11.14-13.78) | 14.44 | (12.95-15.90) | 16.69 | (14.97-18.37) | 19.25 | (17.23-21.21) |
| Unvaccinated | 11.03 | (8.01-13.94) | 12.78 | (9.93-15.54) | 14.80 | (11.96-17.55) | 17.10 | (13.87-20.20) | 19.71 | (15.52-23.70) |
| Nurse-Physiotherapist-Technician/Female | | |  |  |  |  |  |  |  |  |
| Vaccinated after 40 days | 3.86 | (3.25-4.45) | 4.50 | (3.87-5.12) | 5.25 | (4.55-5.94) | 6.12 | (5.29-6.94) | 7.12 | (6.07-8.16) |
| Vaccinated after 60 days | 4.55 | (3.90-5.19) | 5.30 | (4.61-5.99) | 6.18 | (5.41-6.94) | 7.20 | (6.29-8.10) | 8.38 | (7.24-9.49) |
| Vaccinated after 133 days | 7.54 | (6.69-8.39) | 8.77 | (7.83-9.70) | 10.19 | (9.13-11.24) | 11.83 | (10.59-13.04) | 13.70 | (12.23-15.15) |
| Unvaccinated | 7.74 | (5.90-9.53) | 9.00 | (7.38-10.59) | 10.45 | (8.94-11.94) | 12.12 | (10.35-13.86) | 14.04 | (11.48-16.53) |
| Healthcare assistant /Male |  |  |  |  |  |  |  |  |  |  |
| Vaccinated after 40 days | 6.86 | (5.59-8.11) | 7.99 | (6.61-9.34) | 9.29 | (7.74-10.80) | 10.78 | (8.96-12.57) | 12.51 | (10.27-14.68) |
| Vaccinated after 60 days | 8.07 | (6.74-9.38) | 9.38 | (7.94-10.81) | 10.9 | (9.27-12.50) | 12.64 | (10.73-14.50) | 14.63 | (12.31-16.88) |
| Vaccinated after 133 days | 13.22 | (11.63-14.78) | 15.29 | (13.54-17.01) | 17.66 | (15.69-19.59) | 20.35 | (18.09-22.56) | 23.39 | (20.74-25.95) |
| Unvaccinated | 13.55 | (9.13-17.75) | 15.67 | (11.15-19.97) | 18.09 | (13.23-22.69) | 20.84 | (15.22-26.09) | 23.94 | (17.01-30.29) |
| Healthcare assistant /Female |  |  |  |  |  |  |  |  |  |  |
| Vaccinated after 40 days | 4.78 | (7.23-10.33) | 5.58 | (4.65-6.50) | 6.50 | (5.44-7.54) | 7.56 | (6.31-8.80) | 8.80 | (7.23-10.33) |
| Vaccinated after 60 days | 5.64 | (8.69-11.94) | 6.57 | (5.58-7.55) | 7.64 | (6.52-8.75) | 8.89 | (7.56-10.20) | 10.33 | (8.69-11.94) |
| Vaccinated after 133 days | 9.31 | (14.83-18.68) | 10.81 | (9.57-12.04) | 12.54 | (11.12-13.93) | 14.52 | (12.87-16.13) | 16.78 | (14.83-18.68) |
| Unvaccinated | 9.55 | (12.6-21.54) | 11.09 | (8.28-13.81) | 12.85 | (9.84-15.77) | 14.88 | (11.31-18.30) | 17.19 | (12.60-21.54) |

p: percentage cumulative probability of infection estimated from the Cox regression model; 95% CI: 95% Confidence Interval; Rank 1 corresponds to the highest vaccination coverage velocity (days required for 65% of each Healthcare Workers category to complete the vaccination cycle, 39 days), Rank 2 (41 days), Rank 3 (54 days), Rank 4 (67 days), while Rank 5 corresponds to the lowest vaccination coverage velocity among Local Health Authorities (69 days).
